# P04 Polyphenolic extracts from fruit by-products as adjuvants to antibiotics in foodborne bacteria

**DOI:** 10.1093/jacamr/dlag102.010

**Published:** 2026-06-26

**Authors:** Bryan Moreno-Chamba, Julio Salazar-Bermeo, María Concepción Martínez-Madrid, Domingo Saura, Manuel Valero, Victoria Lizama, Nuria Martí

**Affiliations:** Instituto Universitario de Ingeniería de Alimentos-FoodUPV, Universitat Politècnica de València, 46022, Valencia, Spain; Instituto de Investigación, Desarrollo e Innovación en Biotecnología Sanitaria de Elche (IDiBE), Edificio Torregaitán, Universidad Miguel Hernández de Elche (UMH), 03202, Alicante, Spain; Instituto Universitario de Ingeniería de Alimentos-FoodUPV, Universitat Politècnica de València, 46022, Valencia, Spain; Instituto de Investigación, Desarrollo e Innovación en Biotecnología Sanitaria de Elche (IDiBE), Edificio Torregaitán, Universidad Miguel Hernández de Elche (UMH), 03202, Alicante, Spain; Instituto de Investigación, Desarrollo e Innovación en Biotecnología Sanitaria de Elche (IDiBE), Edificio Torregaitán, Universidad Miguel Hernández de Elche (UMH), 03202, Alicante, Spain; Instituto de Investigación, Desarrollo e Innovación en Biotecnología Sanitaria de Elche (IDiBE), Edificio Torregaitán, Universidad Miguel Hernández de Elche (UMH), 03202, Alicante, Spain; Instituto de Investigación, Desarrollo e Innovación en Biotecnología Sanitaria de Elche (IDiBE), Edificio Torregaitán, Universidad Miguel Hernández de Elche (UMH), 03202, Alicante, Spain; Instituto Universitario de Ingeniería de Alimentos-FoodUPV, Universitat Politècnica de València, 46022, Valencia, Spain; Instituto de Investigación, Desarrollo e Innovación en Biotecnología Sanitaria de Elche (IDiBE), Edificio Torregaitán, Universidad Miguel Hernández de Elche (UMH), 03202, Alicante, Spain

## Abstract

**Background:**

Antimicrobial resistance (AMR) poses a serious global health challenge, particularly due to foodborne pathogens such as *Staphylococcus aureus* and *Salmonella enterica*, which exhibit biofilm formation and recurrent infections. Conventional antibiotic treatments are increasingly ineffective, creating an urgent need for alternative or complementary antimicrobial strategies. Polyphenols have demonstrated antibacterial activity, but their clinical application is limited by low *in vivo* efficacy and poor tissue accessibility. Current research therefore focuses on their potential to attenuate bacterial virulence and enhance the activity of existing antibiotics, particularly in epithelial environments. Fruit by-products represent an abundant and sustainable source of polyphenols and may offer novel antibacterial agents with adjuvant potential.

**Objectives:**

This study investigated the antibacterial mechanisms of polyphenolic extracts derived from grape (GBE), strawberry (SBE), and cherry (CBE) by-products against *S. aureus* and *S. enterica*, with particular emphasis on identifying molecular targets and synergistic effects with ampicillin.

**Methods:**

MICs were determined, and bactericidal or bacteriostatic activity was evaluated using time-kill assays. Membrane damage was assessed using CFDA-SE fluorescence, while interactions with bacterial phospholipids were examined through disc-diffusion assays. Synergistic interactions between polyphenolic extracts and ampicillin were also evaluated.

**Results:**

The SBE sample showed the highest inhibitory effect against both strains tested (0.38 mg/mL), while the CBE extract showed inhibitory potential in high doses (12 mg/mL). A bacteriostatic effect of CBE was observed in both strains, while GBE showed a noted bactericidal effect (*P<*0.05). No significant differences in the death rate were identified among samples (*P>*0.05). Fluorescence in cells after polyphenolic exposition revealed that GBE caused cell membrane damage, but not as robust as CBE and SBE, especially when compared to positive control of kanamycin (*P<*0.001). These results suggested that the extracts interacted directly with the cell membrane of bacteria, fact corroborated by phospholipid interactions, where the antibacterial effect of the extracts was lost under cardiolipin presence (*P<*0.05). Finally, the extracts at managed to exert synergism with AMP at 1×, ½× and ¼× MIC of extracts, but CBE, which effect was less prominent.

**Conclusions:**

Overall, fruit-derived polyphenols act as non-traditional antimicrobial agents by targeting bacterial cell membranes and enhancing antibiotic efficacy. These findings support their potential use as dietary-derived antibacterial adjuvants against *S. aureus* and *S. enterica*. Further research is needed to characterize extract composition, clarify interactions with other bacterial cell envelope components, and confirm synergistic effects in more complex biological systems.

